# Oligomerization Interface of RAGE Receptor Revealed by MS-Monitored Hydrogen Deuterium Exchange

**DOI:** 10.1371/journal.pone.0076353

**Published:** 2013-10-01

**Authors:** Ewa Sitkiewicz, Krzysztof Tarnowski, Jarosław Poznański, Magdalena Kulma, Michal Dadlez

**Affiliations:** 1 Institute of Biochemistry and Biophysics, Polish Academy of Sciences, Warszawa, Poland; 2 Institute of Genetics and Biotechnology, Biology Department, Warsaw University, Warszawa, Poland; Cleveland Clinic Lerner Research Institute, United States of America

## Abstract

Activation of the receptor for advanced glycation end products (RAGE) leads to a chronic proinflammatory signal, affecting patients with a variety of diseases. Potentially beneficial modification of RAGE activity requires understanding the signal transduction mechanism at the molecular level. The ligand binding domain is structurally uncoupled from the cytoplasmic domain, suggesting receptor oligomerization is a requirement for receptor activation. In this study, we used hydrogen-deuterium exchange and mass spectrometry to map structural differences between the monomeric and oligomeric forms of RAGE. Our results indicated the presence of a region shielded from exchange in the oligomeric form of RAGE and led to the identification of a new oligomerization interface localized at the linker region between domains C1 and C2. Based on this finding, a model of a RAGE dimer and higher oligomeric state was constructed.

## Introduction

The receptor for advanced glycation end products (RAGE) is a multi-ligand cell surface receptor and member of the immunoglobulin superfamily, first identified by its ability to bind the glycoxidized form of albumin [1]. RAGE is also known to bind other ligands, such as the Aβ peptide, which is implicated in the pathogenesis of Alzheimer’s disease [2,3], the S100 family of proinflammatory cytokine-like mediators [4], Mac1 integrin [5], other amyloidogenic peptides [6–8], the DNA-binding protein amphoterin [9], lipopolysaccharides [10], and phosphatidyloserine [11]. RAGE is an important mediator of the proinflammatory response involved in diverse pathophysiological states, such as neurological disorders, including Alzheimer’s disease [3,12,13], stroke [14,15], amyloidosis [7], the immune response, diabetes, inflammatory disorders [16], infectious diseases [17], and tumors [18].

The binding of RAGE by extracellular ligands activates multiple signaling pathways dependent on the ligand, environment, and cell type. This activation results in the subsequent activation of several transcription factors, including nuclear factor NF-κB, which leads to a proinflammatory cascade and production of pro-inflammatory cytokines, chemokines, and adhesion molecules. Also, due to the presence of NF-κB transcriptional elements in its gene promoter [19,20], RAGE activation causes its own sustained over-expression, converting a transient proinflammatory response into a chronic proinflammatory signal [21]. Such positive feedback loop is a potential therapeutic target, and RAGE has been suggested as a biomarker and/or target for intervention in several diseases, including diabetes and its complications [22,23], inflammation [21], tumors [18] and neurodegeneration [2]. Rational design of therapeutic RAGE antagonists or modulators of RAGE activity requires sound structural knowledge of the receptor itself, its structural forms, and its complexes with ligands. Despite much effort, structural information about RAGE and its interactions with ligands is very limited, and structural aspects of RAGE biology are far from being elucidated fully.

RAGE contains an extracellular region (exRAGE) consisting of three immunoglobulin-like regions, one V-type (amino acids 23-118) followed by two C-type: C1 spanning positions 121-231 and C2 spanning positions 241-319. This region is followed by a relatively long linker and single-pass transmembrane region (tmRAGE, amino acids 343-363) and a 43-amino acid cytoplasmic tail (ctRAGE, amino acids 364-404) [24]. The structure of the full exRAGE has not yet been solved, despite considerable efforts. Until now, only the separate single or tandem domains (V, VC1, C2) have been characterized in detail, by either crystallography (VC1 domain [25] deposited as 3CJJ [26], and MBP-VC1 structure deposited as 3O3U) or nuclear magnetic resonance (NMR) (V domain [27,28] deposited as 2E5E and 2L7U, respectively and C2 domain deposited as 2ENS but unpublished).

Initial NMR studies of exRAGE and its different fragments [29,30] failed to solve the structure directly. The collected data indicate a highly dynamic character of the protein, precluding NOE signal collection. A more stable structure of the isolated V domain was induced and solved in the presence of sodium sulfate, a Hoffmeister series salt known to strengthen hydrophobic interactions [27]. The resulting 20 best structures show the expected IgG fold in which only part of the molecule retains a well-defined structure with large fragments forming several flexible loops (L2, L6, L3; see Figure 5 in ref[27]. , or strands 1-1, 1-2, 1-3, and L3 in ref[30].). A comparison between existing crystallographic and NMR-based structures of the V domain reveals similarities in the regions well-defined in NMR (sequence positions 23-36 and 76-96) and discrepancies in regions found in NMR studies to be flexible and characterized by poor NOE values and signal broadening [27]. These differences indicate the existence of large regions of poorly defined structure in solution. In addition, the kinetics of hydrogen-deuterium exchange in the V domain [27] and exRAGE [31] have revealed generally fast rates of exchange with large, but well-defined, regions of very fast exchange with full flexibility that correlated with the regions indicated as flexible in NMR. These data indicate a high level of plasticity of the large fragments of the exRAGE structure, which may explain the ability to bind diverse ligands [32]. RAGE has been named a pattern recognition receptor [5] by analogy with other receptor systems that recognize common structural patterns shared by different classes of ligands.

The C1-C2 linker is known to be fully flexible [26,29,31], in contrast with the stiff V-C1 linker, which provides the structural coupling between the V and C1 domains, forming an integral structural unit [29]. Thus, exRAGE consists of two structurally independent subunits, VC1 and C2. The majority of ligands bind to the V or C1 domains or both, with the V domain being the main binding site [4]. Advanced glycation end products bind only to the V domain [30,33], S100 proteins to V, C1, or both [4,25,29,34,35], with the exception of S100A6, which also engages the C2 domain [4]. A recent NMR-based structure of the complex of glycated peptide with the V domain revealed that negatively charged ligand residues are electrostatically recognized by a positively charged V domain surface patch. A series of peptide ligand backbone contacts have also been described [28].

Information about ligand-binding events needs to be transmitted in a ligand-specific manner across the membrane, as ctRAGE is known to bind its effector protein in a ligand-dependent way [36,37]. On the other hand, structural uncoupling of VC1 and C2 domains remains unperturbed by ligand binding [29]. Therefore, signal transduction is not likely to propagate across the membrane via allosteric changes within the RAGE domains of the monomeric form of the receptor. Current models do not provide a precise mechanistic answer for how ligand binding to distal domains can be converted to intracellular signal propagation.

The requirement of receptor oligomerization has been proposed as a general mechanism [29,32] to explain signal transduction in RAGE. RAGE oligomerization is likely, as even unligated exRAGE forms constitutive multimers *in vitro* [25,34,35,38]. More importantly, constitutive RAGE oligomers have been detected in the membrane of HEK cells [30,39], even in the absence of ligand binding. Oligomerization seems to precede ligand binding and signal transduction. Ligand introduction is presumed to lead to a shift in the oligomeric form distribution to higher order oligomerization states [25]. Pre-existing oligomerization increases the number of binding sites, and the majority of RAGE ligands have a tendency to oligomerize (Aβ, other amyloidogenic peptides, and S100 oligomers), increasing the stability of the ligand-receptor complex. However, the pathway linking oligomerization and ligand-specific binding of known intracellular effectors, such as ERK [37] Diaphanous-1 [40,41], or TIRAP, and MyD88 adaptor proteins for Toll-like receptors [42] has not yet been elucidated, apart from the conclusion that it increases the colocalization of ctRAGE with the proteins mentioned above.

We decided to continue this line of research by studying the structural consequences of exRAGE oligomerization. We used a model system in which the dimeric exRAGE form is stabilized by a covalent link. We employed hydrogen deuterium exchange (HDex) coupled with mass spectrometry (MS) as an established tool for mapping protein structure and dynamics [43]. We identified a region of RAGE that becomes additionally protected from exchange upon oligomerization, thus defining a new oligomerization interface.

## Results

### Preparation of covalently stabilized oligomeric forms of exRAGE

**Figure 1:**
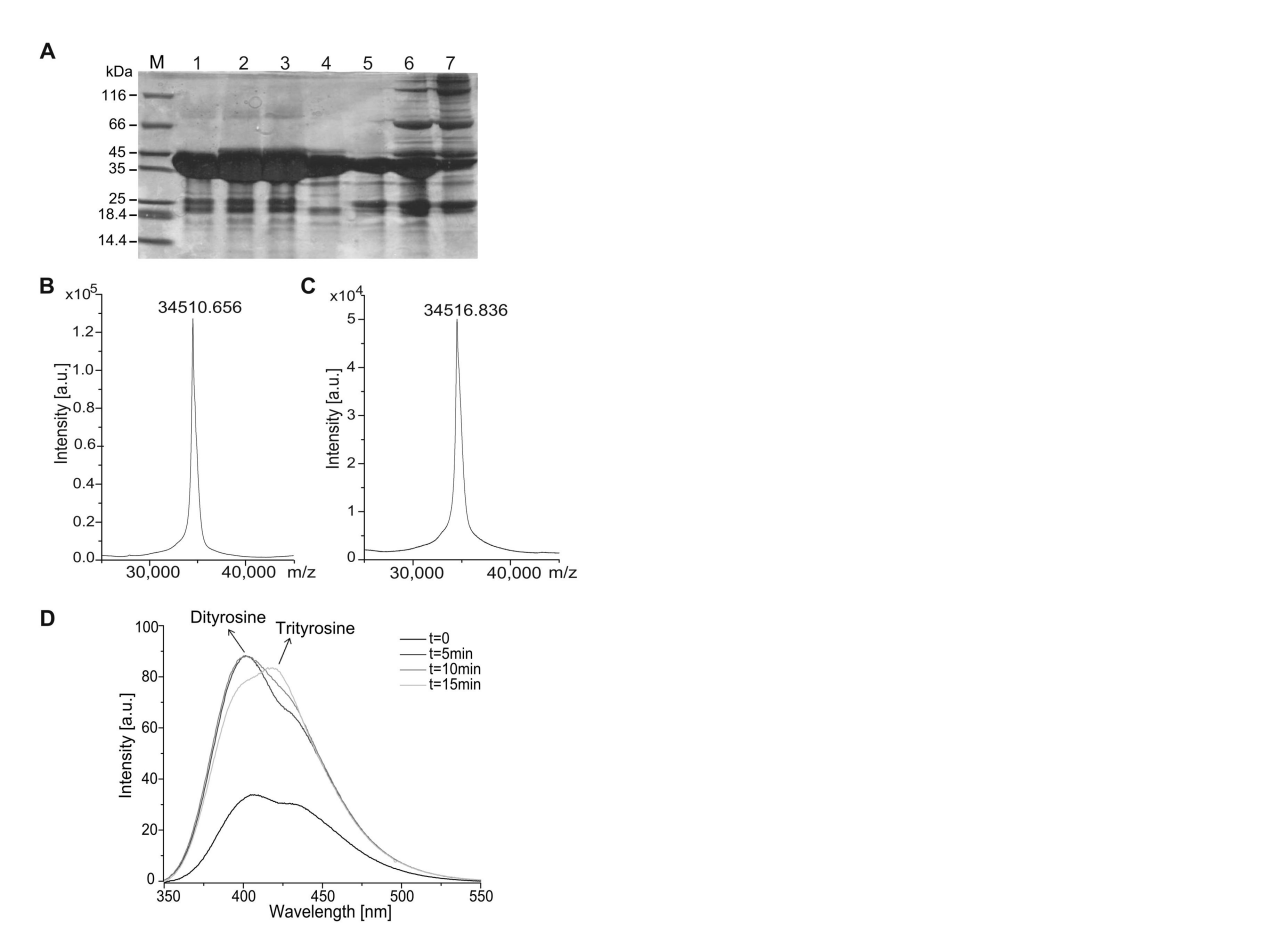
YexRAGE dimer formation *via* a dityrosine cross-link. (**A**) PAGE and (**B**, **C**) mass spectra of exRAGE (**A**, lanes 1-4, B, C) and YexRAGE (**A**, lanes 5-7) incubated with horseradish peroxidase in the presence of hydrogen peroxide for 15 or 30 minutes (lanes 2 and 6 and lanes 3 and 7, respectively). The oligomeric forms visible in lanes 6 and 7 with a mass >45 kDa correspond to YexRAGE dimers and trimers covalently linked by dityrosine and trityrosine bonds, respectively. Such oligomeric forms are absent if the substrate of the reaction is exRAGE instead of YexRAGE (lanes 2 and 3). Lanes 1 and 5 represent the sample before reaction, lane M is a protein ladder, and lane 4 shows the monomeric fraction after SEC. Gels were overloaded on purpose to show the lack of oligomeric forms in the reaction with exRAGE. (**B**) MALDI-ToF mass spectra of exRAGE before and (**C**) after HRP/H _2_O_2_ incubation. (**D**) Fluorescence emission spectra in the range 350-550 nm obtained during incubation of YexRAGE in the presence of hydrogen peroxide and horseradish peroxidase for a specified period of time after excitation at 315 nm. The band with a maximum at 402 nm is expected for dityrosine species, whereas the band at 419 nm represents trityrosine or tetratyrosine species. In the course of the reaction, the fraction of species yielding a signal at 419 nm compared to a band at 402 nm increases, indicating facile formation of multityrosine species in YexRAGE. See also Scheme S1.

Oligomeric forms of exRAGE were obtained using an exRAGE variant (YexRAGE) in which tyrosine residues introduced by substitution of T340Y at the last C-terminal position of the exRAGE sequence were linked by an intermolecular dityrosine (DT) bond (Scheme **S1**). Dityrosine formation was carried out by enzymatic oxidation by horseradish peroxidase (HRP) in the presence of H_2_O_2_. The course of the reaction was followed using polyacrylamide gel electrophoresis (Figure **1A**). Before the reaction, a strong band corresponding to monomeric YexRAGE and much weaker bands corresponding to degraded species were detected. After the onset of oxidation and 30 minutes into the reaction, the monomeric band became weaker and new bands appeared corresponding to dimeric YexRAGE (dimRAGE), trimeric YexRAGE (triRAGE), and possibly tetrameric (tetRAGE) YexRAGE. However, when exRAGE was used instead of YexRAGE, no oligomeric forms were present, even after 30 minutes of reaction, clearly indicating that Y340 is required for DT bond formation. The exRAGE sequence contains four tyrosines at positions 113, 118, 150, and 299, but under the mild reaction conditions used here these tyrosines did not form DT bonds due to being buried inside the protein structure. In the molecular mass spectrum of exRAGE subjected to HRP/H _2_O_2_ treatment (Figure **1B,C**), the signal did not change its shape and position within the precision limits of the mass spectrometer. In addition, a band corresponding to exRAGE after HRP/H _2_O_2_ incubation was excised from the gel and subjected to LC-MS/MS (liquid chromatography mass spectrometry) analysis using an Orbitrap Velos mass spectrometer (data not shown). The data were evaluated for post-translational modifications. No change in the peptide pattern was found for exRAGE after HRP/H _2_O_2_ treatment. These results confirm the homogeneity of exRAGE under the reaction conditions.

Upon dityrosine formation, a characteristic UV absorption/emission band was expected to appear. When YexRAGE was subjected to HRP/H _2_O_2_ treatment, the kinetics of the reaction was monitored by observing changes in the fluorescence signal at 402 nm (Figure **1D**). No such signal was observed when exRAGE was subjected to the reaction (data not shown). Interestingly, at longer reaction times with YexRAGE, the signal at 402 nm was dominated by the shoulder at 419 nm. This finding is in agreement with the appearance of triRAGE and tetRAGE with further oxidation of dityrosine to trityrosine and tetratyrosine. For trityrosine, the maximum emission band is shifted to a longer wavelength [44]. This experiment confirms that the triRAGE form is linked by a trityrosine bridge. The formation of multityrosine bridges was shown previously [45–47].

**Figure 2:**
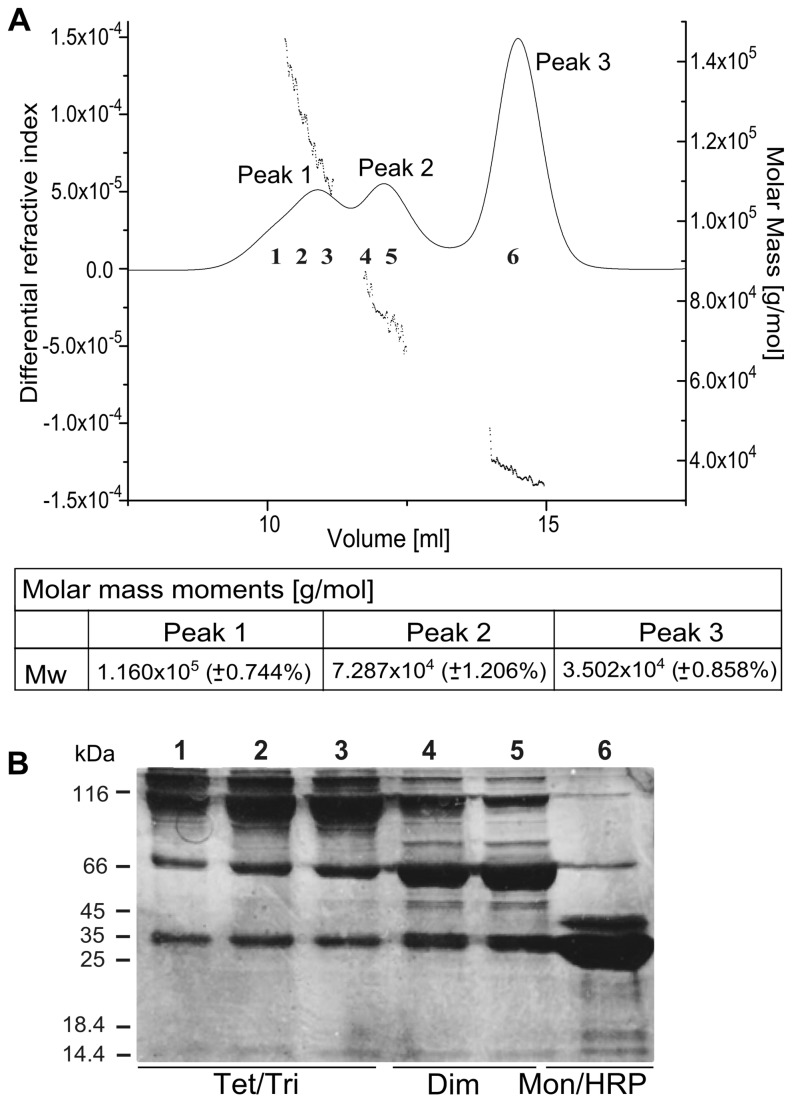
Oligomeric fractions of YexRAGE can be separated by SEC. (**A**) SEC fractionation of YexRAGE after HRP/H _2_O_2_ incubation coupled with MALS mass measurement and (**B**) PAGE of separated species. SEC peaks in (**A**) were detected by the differential refractive index (left axis) with molar masses measured (right axis) for separated species. Average molar masses corresponding to peaks 1-3 are in the lower panel, showing good agreement with the expected mass of YexRAGE monomer, dimer, and trimer. Six fractions were collected as indicated by numbers 1-6 and analyzed by PAGE as shown in (**B**) lanes 1-6. Enrichment in oligomeric species is observed in lanes 1-5.

The molecular masses of the RAGE forms after the reaction were measured by light scattering after separation by gel filtration (SEC) (Figure **2A**). The mass for peak 3 corresponded well to the monomeric species. RAGE at a concentration below 1 mg/ml (~28 µM) was shown previously [48] to be monomeric. Molecular masses corresponding to the other peaks agreed well with the expected masses of the dimer (69 kDa) and trimer (104 kDa). The tetrameric species was not well resolved, forming a shoulder of the trimeric peak. The contents of different SEC fractions were resolved by SDS-PAGE (Figure **2B**), which shows that fractions enriched in different oligomeric forms (dimRAGE and tri/tetRAGE species) can be separated using SEC.

**Figure 3:**
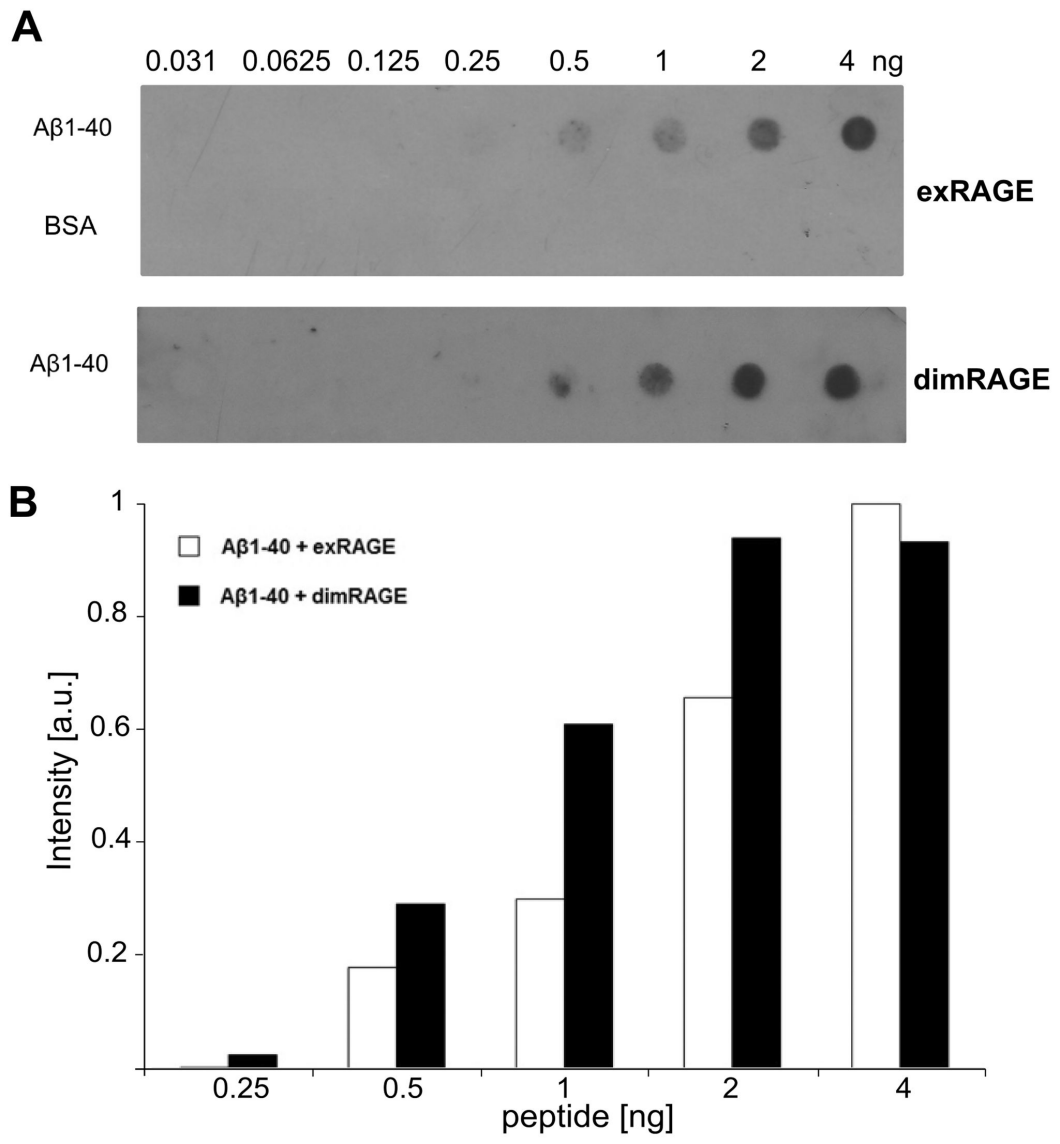
DimRAGE is able to bind a ligand. (**A**) Dot blot showing concentration-dependent binding of exRAGE (upper panel) and dimRAGE (lower panel) to Aβ peptide. (**B**) Densitometry illustrating retained binding of dimRAGE to Aβ peptide, one of the ligands of RAGE. When BSA was used as the negative control, no binding was observed.

We used a dot blot assay to ensure that dimRAGE retains the ability to bind ligands (Figure **3**). Both exRAGE and dimRAGE were detected in a concentration-dependent manner when the test was carried out using Aβ-peptide-soaked blotting paper. Thus, dimRAGE has comparable binding properties for one of its ligands compared to exRAGE.

By covalently linking exRAGE C-termini at position 340, we obtained a functional dimer of exRAGE. Position 340 marks the start of the ctRAGE region and is 19 amino acids from the C-terminal residue (I321) involved in the C2 domain structure. Such a long, flexible (containing seven glycine residues) linker ensures the flexibility of the mutual positioning of the two RAGE monomers in dimRAGE. A dityrosine “molecular staple” mimics the anchoring of the exRAGE C-terminus in the membrane and provides entropic stabilization of a homo-oligomer, allowing insight into the structural consequences of oligomerization.

### Hydrogen-deuterium exchange analysis

The pattern of hydrogen-deuterium exchange (HDex) for the dimRAGE SEC fraction was compared to the pattern of its monomeric counterpart. An advantage of the MS-based HDex method is that the experiment is carried out at a low protein concentration (<10 µM), for which the intermolecular interactions are relatively destabilized, at least compared to NMR or crystallography. Thus, our approach provides an opportunity to compare oligomeric states with a predominantly monomeric population of RAGE. RAGE was monodisperse only at relatively low concentrations (<28 µM); higher concentrations, as required for NMR, may lead to excessive intermolecular contacts and mask the monomeric behavior.

As the first step of measuring HDex pattern, the LC retention times and ion mobility drift times of the peptic peptide sequences of YexRAGE were obtained by LC-MS-MS/MS analysis of the undeuterated sample. The peptide list obtained using PLGS software is provided in Table S1. The peptides from the list covered 76% of the exRAGE sequence.

Analysis of the HDex pattern of the dimRAGE SEC fraction (experiment D) was accompanied by three control experiments (A, B, and C) in which three different variants of RAGE were subjected to analysis: experiment A, untreated exRAGE; experiment B, exRAGE subjected to HRP/H _2_O_2_ treatment; experiment C, untreated YexRAGE. For each experiment, two additional technical control analyses were conducted as described previously [31]. Briefly, in the first analysis, the out-exchange control, the exchange level (m/z${}_{\text{ex}}^{\text{100}}$) was measured in the peptic peptides after prolonged incubation in D_2_O to ensure their complete exchange. These values represent the maximum (100%) possible m/z_ex_ value that can be obtained in a given experimental setup for a given peptide. The minimal (0%) level of exchange was measured in the second control experiment, the in-exchange control, which allowed the measurement of the unavoidable exchange level when the protein was loaded on the pepsin column at low pH and low temperature dissolved in the buffer containing D_2_O. The m/z${}_{\text{ex}}^{\text{0}}$values obtained in this experiment represented the minimum m/z_ex_ value that could be obtained in the experimental setup and represented the lack of exchange (0%) control. In the conditions of our present experiment, the in-exchange level was observed to be very low, and in some cases negligible. The experimental scheme allowed us to detect the same set of peptides in control experiments and the exchange experiment. The two control values, m/z${}_{\text{ex}}^{\text{0}}$ and m/z${}_{\text{ex}}^{\text{100}}$, were used to calculate the fraction of exchange.

**Figure 4:**
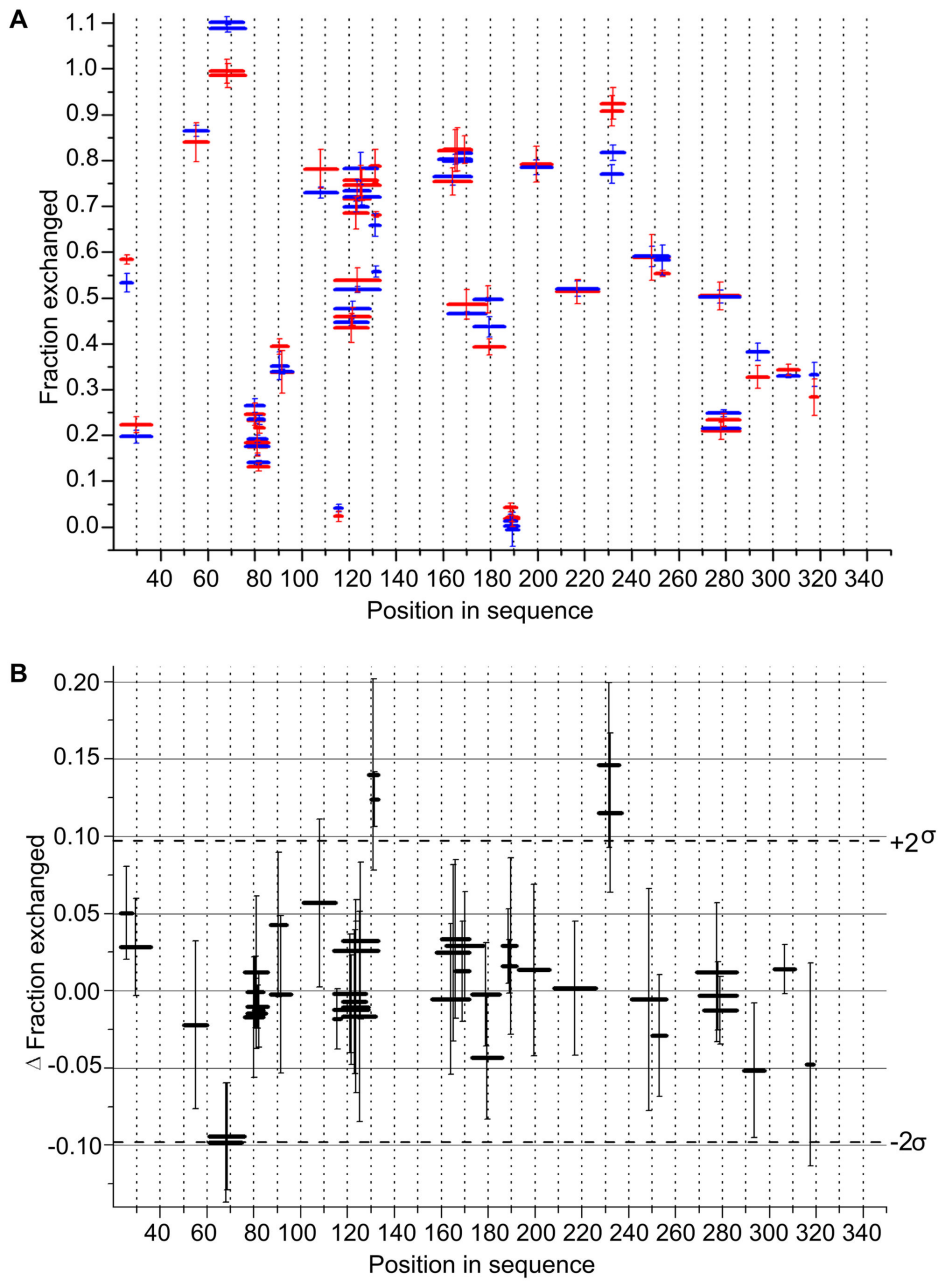
Significant differences in the pattern of exchange for exRAGE and dimRAGE. (**A**) Comparison of the fraction of amide protons of exRAGE (red) and dimRAGE (blue) exchanged after 10 seconds. The position of a peptide is shown on the horizontal axis, represented by a horizontal bar with a length equal to the length of the peptide. Marks of the same length centered at the same x-axis value but with a slightly different fraction of exchange denote the data obtained for a different charge state of the same peptide. Small differences in the exchanged fraction for different charge forms or overlapping peptides underscore the good internal consistency of the data. Y-axis error bars are standard deviations calculated from at least three independent experiments. For a large majority of peptides the level of exchange did not differ significantly between exRAGE and dimRAGE and retains intertwining between relatively protected and completely unprotected regions observed previously [31]. However, significant differences between exRAGE and dimRAGE were detected at two protein regions represented by four peptides. For better visualization of these differences (**B**) shows the result of subtracting (Δ) the fraction of exchange in exRAGE and dimRAGE with positive Δ values indicating increased protection in dimRAGE. Error bars were calculated by summing the standard deviation values of the subtracted data points. Two peptides in the region centered at position 131 and two centered at position 231 show increased protection by more than 2σ upon transition from the monomeric form to the oligomeric form. See also Figure S1.

**Figure 5:**
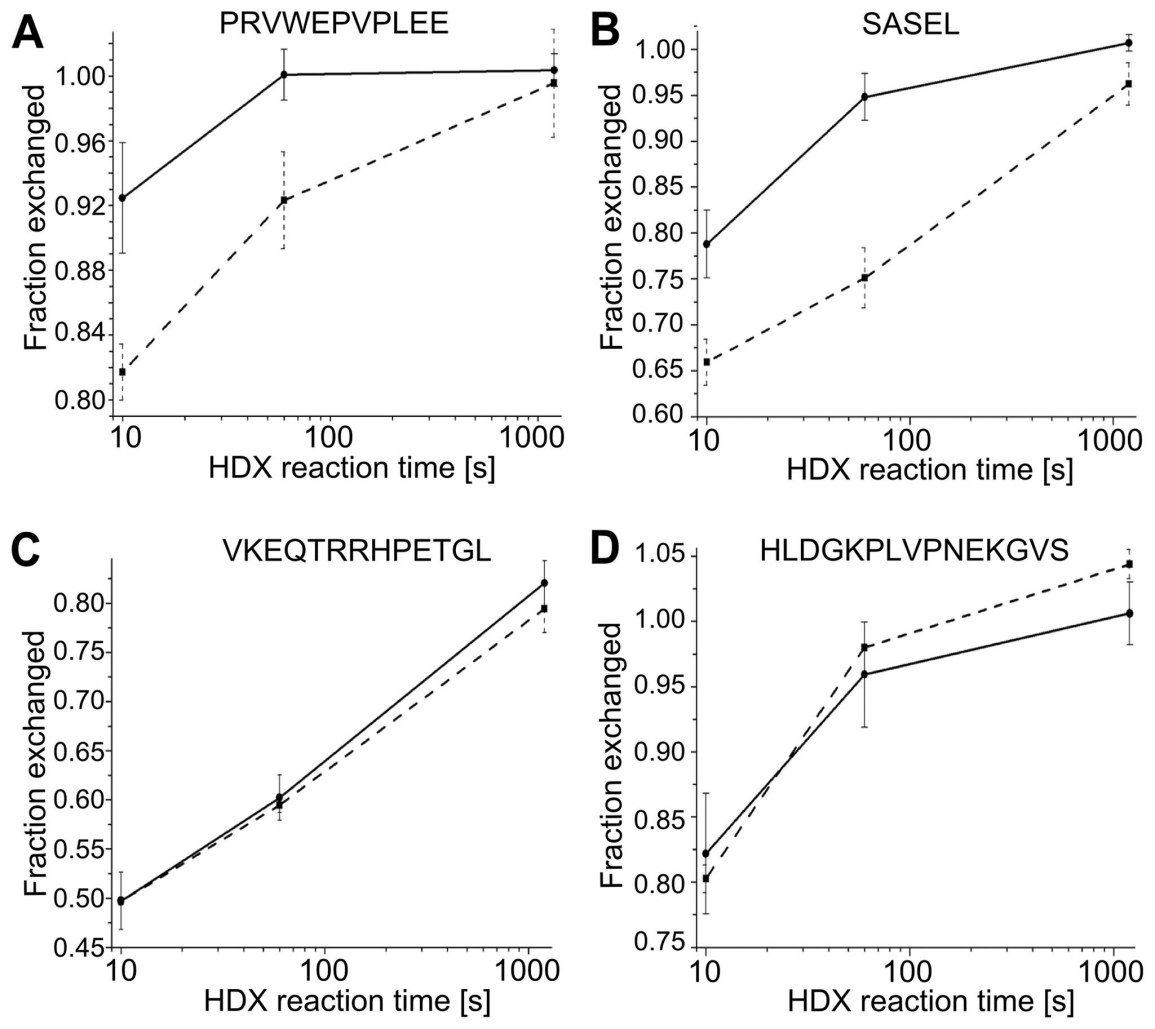
Kinetics of HD exchange in four selected exRAGE peptides. (**A**) PRVWEPVPLEE, (**B**) SASEL, (**C**) VKEQTRRHPETGL, and (**D**) HLDGKPLVPNEKGVS fraction of exchanged proteins was measured for exRAGE peptides were measured three times after incubation in D_2_O for 10 seconds, 60 seconds, and 1200 seconds and shown on a logarithmic timescale. Filled circles and lines represent data for monomeric RAGE, whereas filled rectangles and dashed lines represent data for dimRAGE. Significant differences were observed in the fraction of exchanged peptide amides at shorter incubation times in (**A**) and (**B**) only. Error bars are standard deviations calculated based on at least three experiments. See also Figure S2.

The general pattern of exchange characteristic of exRAGE [31] was strictly retained in all three control experiments, and the difference in the fraction exchanged after mutation or exRAGE incubation with HRP/H _2_O_2_ did not exceed a few percent (Figure **S1A,B**). The T340Y mutation or subjecting exRAGE to HRP/H _2_O_2_ treatment did not change the exchange pattern, indicating a lack of structural changes. However, when the pattern of exchange for monomeric RAGE was compared with the pattern of exchange for dimRAGE, reproducible and significant differences were observed in selected regions of the protein. Increased protection upon oligomerization was detected after 10 seconds of exchange in four peptides (Figure **4A,B**), hereafter referred to as differential peptides. The kinetics of exchange for two differential peptides, and two other peptides for comparison, are shown in Figure **5**. The observed differences were stronger after incubating for 10 seconds, and significant at 1 minute, whereas the exchange was close to completion at 20 minutes in all cases. Figure **S2** shows the isotopic envelopes corresponding to the two selected RAGE peptides.

**Figure 6:**
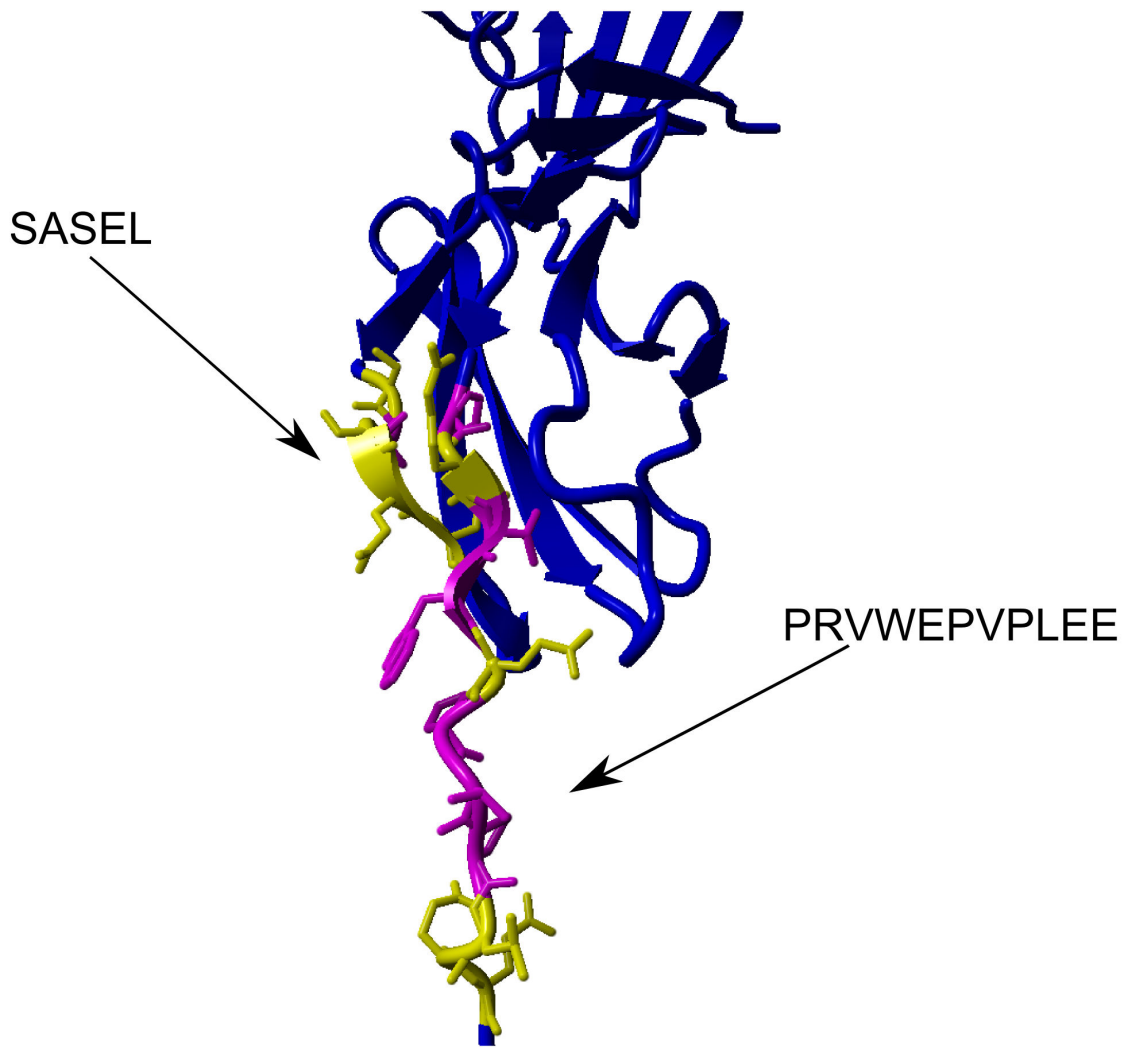
Localization of protected peptides in the exRAGE dimer in VC1 structure 3CJJ (ribbon representation). Regions 129-133 (SASEL) and 227-237 (PRVWEPVPLEE) are highlighted in magenta (hydrophobic residues) and yellow (other residues). See also Figure S3.

Two of the differential peptides, ASEL and SASEL, cover regions 130-133 and 129-133, respectively, and two other peptides, PRVWEPVPLE and PRVWEPVPLEE, cover regions 227-236 and 227-237 of the RAGE sequence, respectively. The latter peptides contain sequence P_227_RVW_230_ from the C-terminal portion of strand 4-2 in VC1 domain structure 3CJJ and the E_231_PVPLEE_237_ fragment corresponding to the C1-C2 domain linker region (C2 domain 1-1 strand starts at position 241). In monomeric exRAGE, the C1-C2 linker is flexible and unprotected from exchange [31], so our data indicate that the partial protection in the linker region observed here originates from oligomerization. When overlaid on VC1 structure 3CJJ, all four peptides localized to a single beta sheet consisting of strands 4-1 and 4-2 and the C1 to C2 linker region. Figure **6** shows the placement of residues belonging to these peptides in the 3CJJ structure, underscoring the fact that 9 out of 14 residues in region 227-240 are largely exposed hydrophobic residues: three valines, two leucines, three prolines, and one tryptophan. Shielding of the hydrophobic surface in the oligomer is expected to be favorable thermodynamically. This group of residues forms a plausible contact surface for dimer formation, involving the residues of C1 domain β-sheet 4 and part of the C1-C2 linker. Moreover, no changes were detected in any other region of exRAGE, which suggests that the rest of the structure is not affected by or involved in the dimerization process. Because only a single binding interface was indicated, the same region in both monomers is involved in dimer formation.

### Molecular modeling of the dimer

**Figure 7:**
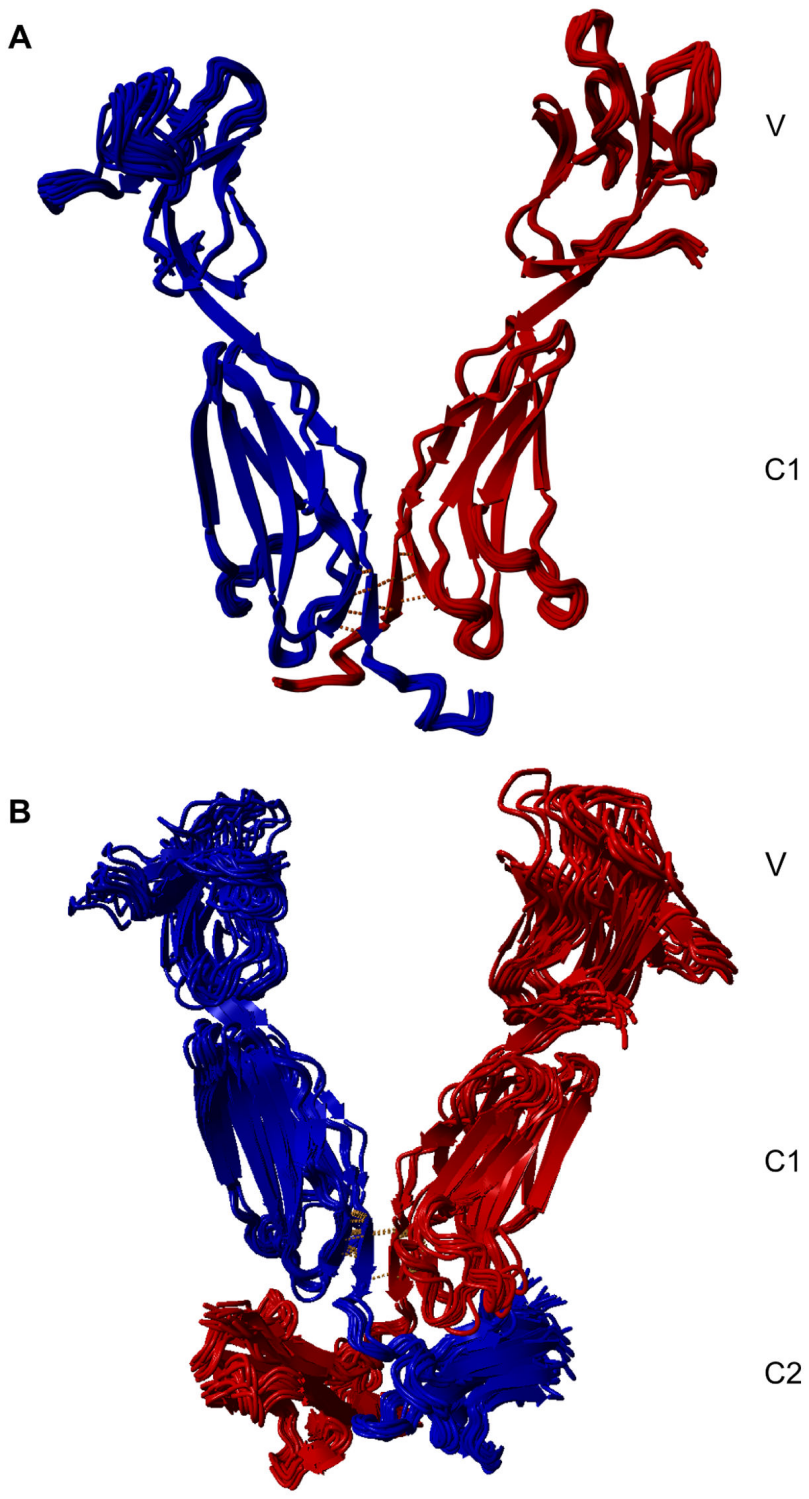
Molecular model of the RAGE dimer. (**A**) Dimer constructed by molecular modeling of the VC1 3CJJ structure rotated by 180°. (**B**) Dimer from (**A**) with the C2 domain appended. Twenty snapshots from the last 20 ns of molecular dynamics are overlaid and two RAGE monomers marked with different colors. In (**A**), the C1-C2 linker residues become positioned in the swapped arrangement due to interactions with E-F loop of the C1 domain of the second molecule. When the C2 domain is appended in (**B**), the swapped arrangement of the linker and C2 domain is retained and the C2 domain accommodated well, supported with a set of favorable contacts between the linker and C2 domain residues with C1 domain loops E-F and C’-D. Orange bars mark the network of hydrogen bonds linking two molecules. See also Figure S4.

Observed differences in HDex most likely result from changes in the hydrogen-bonding network [49]. The PRVW peptide in the VC1 structure forms one of the two beta strands of β-sheet 4, which is hydrogen bonded to peptide SASEL on one side, and exposed to solvent on the other side. A molecular model of the RAGE dimer was constructed by pairing a VC1 monomer with the second molecule rotated by 180° into a C2 symmetry dimer. The last 20 snapshots of the molecular dynamics simulations of the dimerized VC1 structure are shown in Figure **7A**. In the model, the hydrogen-bonding network of sheet 4 is strengthened and hydrogen-bonding capacity of peptide PRVW fully saturated. Thus, the structural constraints derived from HDex experiments are accommodated in the model. In addition to saturated hydrogen bonds, the interface engages a series of hydrophobic residues present in the C1-C2 linker sequence **W**E**PVPL**EE**V**Q**L**, which in majority are exposed to solvent in the VC1 monomer. The difference in the solvent accessible surface in the monomer and dimer indicates that approximately 900 Å^2^ of surface buried at the dimer interface is mainly hydrophobic in character, corresponding to roughly 10 kcal/mol of dimer-stabilizing free energy [50]. Interestingly, the profile of contacts between buried residues (Figure **S3**) indicates that C1-C2 linker residues are buried due to contact with P_196_ARGGDPR_203_, corresponding to C1 domain loop E-F of the second monomer [25]. In addition, this contact can be stabilized by an Arg residue salt bridge of the loop with the Glu residue of the linker. In the mode the linker becomes swapped between the monomers in the dimer. The linker region contacts extend to V238 and L240, positioning the first residues of the C2 domain, which starts at V241. To determine whether such linker positioning is compatible with extending the VC1 with C2 domain, we repeated the molecular dynamics simulation of the dimer shown in Figure **7A**, but with an appended C2 domain. The 20 last snapshots of the analysis are shown in Figure **7B**. In the hypothetical VC1C2 dimer model, the C2 domains remained well-positioned in their swapped arrangement, and the swapped contact surface was expanded (~2300 Å^2^ buried surface averaged over 20 snapshots) and enforced by a possible Arg169 (loop C’-D in C1) - Glu243 or Glu245 from C2 salt bridge. Approximately 550 Å^2^ of buried surface was added by the direct contact of the G_309_PQESR_314_ regions of two C2 domains. Due to poor coverage of this region in HDex, this interaction could not be confirmed experimentally. The molecular model of the dimer accommodates the C2 domain well and suggests a hypothetical domain swapping mechanism by which ligand binding can lead to oligomerization-mediated changes in positioning of the C2 domain, enabling ligand-specific signal transduction.

## Discussion

Structural consequences of oligomerization were studied using a covalently stabilized oligomer of RAGE. Oligomeric forms of RAGE are thought to be important mediators of the signal transduction cascade, which leads to sustained pro-inflammatory signaling in multiple diseases. Many receptors are known to be activated by ligand-induced oligomerization, including TNFα receptor, Toll-like receptors, and the PDGF receptor subfamily [51]. Oligomerization of RAGE has been suggested as an alternative that allows the lack of structural coupling between its ligand-binding regions and the intracellular domain to be overcome. Structural constraints obtained in this work from the analysis of HDex patterns indicate a new oligomerization interface localized at the link between the C1 and C2 domains. Dimerization *via* this interface allowed us to build a model of the RAGE dimer, suggesting a new molecular mechanism for the required coupling of an extracellular event and intracellular downstream signaling.

To construct a dimeric form of the receptor, two RAGE molecules were covalently bound by a dityrosine, linking the two tyrosine residues engineered to the C-terminus of the protein construct. Dityrosine cross-links have been detected in many proteins, including crystallins, elastin, and fibroin [52,53]. Cross-linking by induction of dityrosine *via* the peroxidase–hydrogen peroxide system has been reported to be efficient in *in vitro* cross-linking of resilin monomers to obtain a new elastomeric material [54], and it has been suggested to be a versatile tool for protein immobilization [55]. Whenever possible, a DT cross-link is preferable over a classic disulfide cross-link due to its stability. RAGE is stabilized by three disulfide bonds and engineering of an additional cysteine residue may lead to the rearrangement of native disulfides. For these reasons a dityrosine cross-link was used, and the cross-linking reaction was found to be specific and very fast. The reaction may have been facilitated by an entropic mechanism caused by non-covalently stabilized oligomers, which could bring the two tyrosines into relative proximity. Non-covalent oligomerization is not unexpected in the RAGE preparations. A tendency of exRAGE to oligomerize in solution has been documented [34]. The low order oligomers might represent the native uninduced state of the receptor, whereas exposure to ligand “enhances” oligomerization [39] or acts by shifting the equilibrium towards higher order oligomeric states.

Nevertheless, the basic oligomeric state is a dimer and our data indicate a possible dimerization interface involving C1 C-terminal residues and C1-C2 linker residues. Based on this observation, we constructed a molecular model of the rotational VC1 dimer (R-dimer) with C2 symmetry using molecular dynamics. According to this model, monomers contact only *via* a hinge region composed of a four-stranded β-sheet, two strands from each monomer’s β-sheet 4 (Figure **7**). Interestingly, strands 4-1 and 4-2 are ‘additional’ β strands, which is unique for RAGE, and are not present in other immunoglobulin fold proteins [25], making them perfect candidates for RAGE-specific oligomerization. In the model, VC1 domains form V-shaped arms with spatial organization that enables the accommodation of a ligand with a diameter exceeding 40-45 Å. This size roughly corresponds to the dimension of an S100B octamer [35]. Such a V-shaped dimer would allow bi-dentate binding of large ligands, such as glycated albumin, as discussed in Figure 8 in ref[30]. . However, due to small rearrangements of a sheet 4 hinge, a scissor-like arrangement may provide enough flexibility of the VC1 arms in the dimer to accommodate smaller ligands that require a smaller distance between the V-domains. Alternatively, smaller ligands may bind to the internal surface of the VC1 arms. This surface is lined with positively charged residues (Figure **S4**) in accordance with RAGE ligand binding code. The binding of ligands of different sizes or shapes may lead to hinge adjustments and enforce changes in the arrangement of the C2 domain and intracellular domains necessary for ligand-dependent signaling [36,37]. Moreover, the C2 symmetry of the dimer facilitates the docking of C2 symmetry ligands, including dimers of nearly all S100 family members, tetra(octa)mers of S100B, and higher order oligomeric forms of S100B, S100A4, S100A8/A9, and S100A12 [4].

Another interesting feature of the model is swapping of the linker region and the C2 domain. Swapping the linker between monomers in the dimer was suggested previously [25]. An interesting consequence of the positioning of C2 in the model is that it localizes the Ile_321_ residue (C-terminus of C2 domain) distal from the membrane. This position does not prevent anchoring of the molecule in the membrane; residue 321 is separated from the tmRAGE region by 19 amino acids (epgeegptagsvggsglgt) of the flexible linker. Consequently, the model states that, upon formation of the oligomer, the extracellular part of the receptor is brought closer to the membrane. This positioning may have consequences on the efficiency of receptor internalization, which was recently indicated as a necessary step for signal transduction [56].

A different model of the dimer was proposed recently on the basis of the crystallographic structure of the VC1 domains [25]. VC1 molecules in protein crystals present an extended network of intermolecular contacts. The presence of zinc ions is necessary to obtain the crystal, and the structure contains a zinc ion embedded at the intermolecular interface. Zinc has been suggested to be required for dimer formation, along with the pairing of region 176-189 in one molecule with region 158-168 in the second molecule (see Figure 3 in ref[25].). Such a dimer has translational symmetry (T-dimer) similar to some RAGE ligands, such as Aβ oligomers [57].

**Figure 8:**
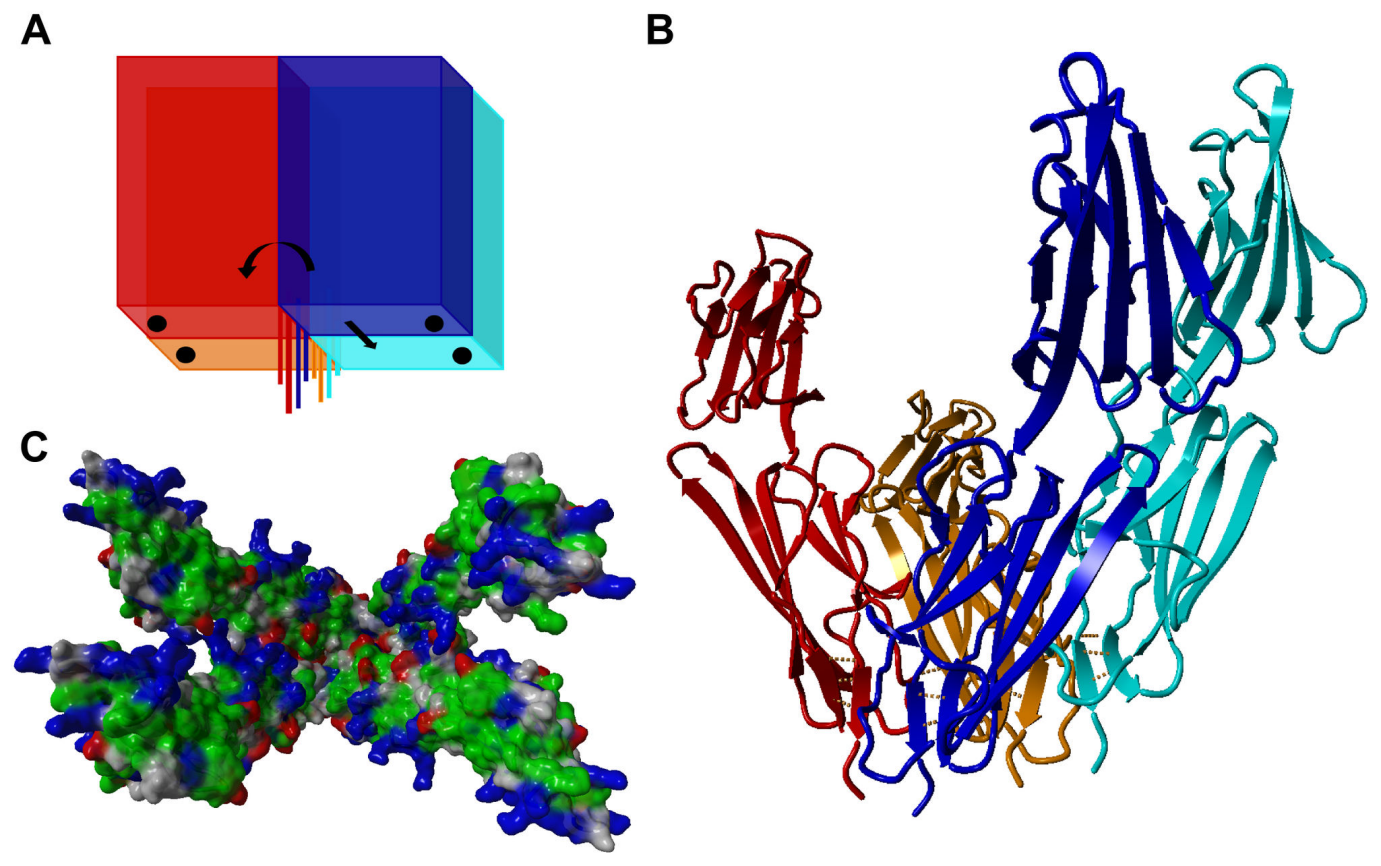
Molecular model of the RAGE tetramer. (**A**) Tetramerization scheme in which the zinc-stabilized dimer from ref[25]. (T-dimer) was rotated and docked, reconstructing a dimer of two C2 symmetry dimers (R-dimers). (**B**) A molecular model was constructed according this scheme and subjected to molecular dynamics. The last 20 snapshots are shown. The model shows good compatibility between two dimeric structures, retaining the network of hydrogen bonds in sheet 4 β-strands linking four molecules, marked by orange bars. The enforced hydrogen bonding in this region explains the stabilization of hydrogen bonds observed for sheet 4 peptides in HDex. (**C**) The tetramer exposes positively charged internal binding surfaces in the form of four VC1 domain arms capable of binding ligands of different sizes and symmetries, either rotational (as S100B tetramer) or translational (as Aβ oligomers).

Interestingly, the C1 domain β-sheet 4 region in the VC1 crystal structure of the dimer [25] is well exposed to intermolecular contacts, so oligomerization mediated by both regions is not mutually exclusive. Both dimer models can be reconciled in a hypothetical model of the tetramer constructed by the rotation of two T-dimers (Figure **8A**). In this model, tetramerization is mediated by the C1 β-sheet 4 region (Figure **8B**). Such a docking experiment shows the compatibility of two T-dimers and two R-dimers integrated into one tetramer coupled by an eight-stranded β-sheet “keyboard” consisting of strands 4-1 and 4-2 from four molecules. The VC1 “arms” remain apart, forming a concave ligand-binding surface exposing large positively charged patches (Figure **8C**) necessary for binding negatively charged ligands. Thus, the constructed tetramer would accommodate ligands oligomerizing with both rotational and translational symmetry. Moreover, the tetramer could propagate into larger oligomers because additional dimers can attach to the tetramer.

In conclusion, we have found that oligomerization of the extracellular part of the receptor leads to structural changes that are localized in the vicinity of the C1-C2 linker region. To accommodate these experimental constraints, we constructed a new model of the dimeric/tetrameric forms of exRAGE. This model explains the HDex results, but also provides a mechanistic explanation of ligand-dependent signal transduction into the cell.

## Materials and Methods

### Protein expression and purification

The exRAGE protein and its variant YexRAGE were over-expressed in bacteria. The expression construct containing a C-terminal His-tag was a gift from Dr. Rosemarie Wilton, Argonne National Laboratory, IL, USA. YexRAGE was obtained by a point mutation at position 340 that changed threonine to tyrosine (more information are found in Text **S1**).

### Dityrosine crosslinking of YexRAGE

Dityrosine crosslinks were generated by incubating YexRAGE with horseradish peroxidase (HRP) and hydrogen peroxide (H_2_O_2_) in a buffer containing 20 mM Tris-HCl and 800 mM NaCl, pH 7.2. The stock solution of YexRAGE was diluted to a final concentration of 200 µM in the above reaction buffer. HRP was dissolved in 20 mM Tris-HCl, pH 7.2. All reagents were freshly prepared or stored no longer than 3 days. The concentration of HRP was determined spectrophotometrically (Cary 50 Bio, Varian, Palo Alto) at 280 nm using a molar extinction coefficient of 39,800 M^-1^cm^-1^. The reaction product was detected fluorometrically using a fluorescence spectrometer (Cary Eclipse, Varian, Palo Alto) at an excitation wavelength of 315 nm, with a characteristic emission band for dityrosine centered at 400-404 nm [58]. Spectra were recorded in 10 mm cuvettes at 350 to 550 nm, 1 nm/s, 25 °C. In a control experiment, exRAGE was incubated with HRP and H_2_O_2_ in 20 mM Tris-HCl and 800 mM NaCl, pH 7.2.

The reaction product was fractionated using AKTA FPLC with a Superdex 200 10/300 GL column (GE Healthcare, Amersham Biosciences) and 20 mM Tris and 800 mM NaCl (pH 7.2) as a mobile phase at 0.5 mL min^-1^. UV absorbance (280 nm) was used for detection.

Dityrosine cross-linked YexRAGE was analyzed by SDS-PAGE to confirm the presence of oligomers. Samples were collected at specified reaction times: 0 min, 15 min, and 30 min. Aliquots from each reaction were heated to 90 °C for 10 minutes in electrophoresis sample buffer containing 4% SDS and 2.5% β-mercaptoethanol. Molecular size marker (Unstained Protein Molecular Weight Marker, Fermentas) and the YexRAGE samples were separated on 4-12% polyacrylamide gels and stained with Coomassie Brilliant Blue.

### Size-Exclusion chromatography with multi-angle light scattering/refractive index detection (SEC-MALS/RI)

We used MALS/RI detection in conjunction with size-exclusion chromatography to determine the molecular weights of oligomers formed by YexRAGE after HRP/H _2_O_2_ treatment. The sample was loaded into the flow cell and separated at 10°C on a Superdex 200 10/300 GL column (GE Healthcare, Amersham Biosciences). MALS and RI were performed with DAWN HELEOS-II MALS and Optilab T-rEX detectors (Wyatt Technology Corp, Santa Barbara, CA), respectively. The laser wavelength was 662.0 nm. The mobile phase used in light scattering experiments was 20 mM Tris and 800 mM NaCl, pH 7.2, at a flow rate of 0.7 mL/min.

Astra software (version 6.0.3, Wyatt Technology Corp, Santa Barbara, CA) was used to analyze the data by means of a Debye plot to generate molecular parameters, including the weight-average molar mass (*M*w) and the *z*-average RMS radius (*rz*). The refractive index was determined using parameters from Optilat-TrEX. The weight average and average molar mass were calculated from the refractive index using the Zimm model.

See **MALDI-TOF mass spectrometric analysis** and **Dot blot assay** in Text **S1**).

#### Hydrogen-deuterium exchange

All experimental procedures used for this method are found in Text **S1**.

### HDex data analysis

During the first stage of the data analysis, the deuteration level for each peptide resulting from exchange was calculated in an automated manner using DynamX software, based on the peptic peptide list obtained from the PLGS program. The list was further filtered in the DynamX program using the following acceptance criteria: minimum intensity threshold 3000, minimum products 2, minimum products per amino acid 0.15, and minimum consecutive products 1. Analysis of the isotopic envelopes after exchange was carried out in DynamX using the following parameters: RT deviation ± 9 s, m/z deviation ± 15 ppm, drift time deviation ± 2 time bins. The values for deuterium uptake in the exchange experiment (M_ex_) and two control experiments (M${}_{\text{ex}}^{\text{0}}$ and M${}_{\text{ex}}^{\text{100}}$) obtained from the automated analysis were verified by visual inspection. Ambiguous or overlapping isotopic envelopes were discarded from additional analysis. The final data were exported to an Excel (Microsoft) spreadsheet for calculation of the HDex mass shifts and the fraction of exchange. The fraction of exchange (f) of a given peptide was calculated by taking into account both control values as follows:

$${\text{f = (M}}_{\text{ex}}{\text{-M}}_{\text{ex}}^{\text{0}}{\text{) / (M}}_{\text{ex}}^{\text{100}}{\text{-M}}_{\text{ex}}^{\text{0}}\text{)}$$

Error bars for the exchange fraction (f) are represented by the standard deviations of at least three independent experiments. For estimation of the error in the difference in exchange (ΔHDex) obtained by subtracting the fraction of exchange in the dimer (f_d_) and monomer (f_m_) (Figure **5**), the simplest model of error propagation was assumed in which ΔHDex errors were calculated by summing the standard deviation values of subtracted numbers (f_d_ and f_m_). Final figures were plotted using Origin (Microcal) software.

### Molecular dynamics analysis

Molecular dynamics (MD) analysis was carried out using the Yasara Model (Yasara website. Available: http://www.yasara.org. Accessed 2013 Sep 4) with standard Yasara two-force field [59]. A computer cluster HPC "Nest" constructed using 80 double-processor servers (128 Intel Xeon X5650 CPUs, 2.67 GHz (6 cores) and 32 AMD Opteron 6174 CPUs, 2.2 GHz (12 cores)) was used. The cluster is organized using a Lustre (Intel) file system constructed on six disk arrays (100 disks including 60 SSD disks in RAID0 configuration) and redundant Infiniband (Melanox) network.

The initial structures were solvated in boxes of water molecules, the dimensions of which allowed for at least 10 Å between the protein and the box border. To preserve the general fold, distance constraints were applied for intramolecular H-bonds identified in PDB structures (3CJJ and/or 2ENS) and dihedral constraints for all residues located in secondary structural elements (e.g., α-helices and β-sheets). In addition, distance constraints defining the topology of hydrogen bonds in the postulated intra-oligomer interface were also added. MD simulations were carried out for an initial 5 ns with fixed backbone atoms to enable tuning the side chains of residues located in the inter-oligomer interface. During the next 10 ns, the system was released to evolve and snapshots taken every 500 ps.

Topology of the C1 dimer was constructed to accommodate the localization of experimentally obtained structural constraints in the form of increased protection of the amide protons of residues 129-133 and 227-237 in the dimer. These residues are located in a two-stranded parallel β-sheet 4 (3CJJ), and propagation of the β-sheet in this region is assumed to accompany protein oligomerization. Heterogeneous parallel-antiparallel β-sheet topologies are rarely reported in PDB records (as identified in the SCOP database, http://scop.berkeley.edu [60], Accessed 2013 Sep 4), and it was assumed to be unlikely. Instead, a four-stranded parallel β-sheet was assumed to be the most likely topological element at the inter-monomer interface. Consequently, due to intermolecular steric restrictions, the most putative intermolecular interface must involve oligomerization along the edges of β-sheet 4.

Detailed analysis of the location of two VC1 molecules in the asymmetric crystal cell on which the previous dimer model was based [25] demonstrated that the two β-sheets 4 are organized in a parallel “keyboard-like” arrangement in the translational symmetry dimer (T-dimer). However, a gap between these two sheets is present in the T-dimer, which could accommodate the next two-stranded β-sheet, saturating a multi-stranded β-sheet (see Figure **8A**). Following this observation, an initial topology of the C1 tetramer was set as a rotational dimer of two T-dimers stitched by a hydrogen bonding network of four two-stranded β-sheet elements coalescing into a one multi-stranded parallel β-sheet.

VC1 dimer was obtained as a projection of the VC1 monomer taken directly from the PDB record of the calculated structure of the C1 dimer by superposition on residues located in β-sheets of the C1 domain. VC1 tetramer was obtained analogously as a projection of the VC1 monomer on the calculated structure of the C1 tetramer. VC1C2 dimer and tetramer were obtained by superposition of the C-terminal portion of the VC1 dimer/tetramer with corresponding N-terminal residues of the C2 monomer (2ENS). All of these structures were subjected to exhaustive MD rounds.

## Supporting Information

Scheme S1 **Mechanism of Dityrosine formation.**
(TIF)

Figure S1 **No differences detected between exRAGE and YexRAGE or exRAGE treated with HRP/H _2_O_2_.** Control comparison of the exchange pattern in exRAGE with (*A*) YexRAGE or (*B*) exRAGE subjected to HRP/H _2_O_2_ treatment. The difference in the exchanged fraction is shown on the vertical axis for peptides represented by horizontal bars of a length equal to the length of the peptide and placed at the appropriate position in the sequence on the horizontal axis. Y-axis error bars were calculated by summing the standard deviation values of the subtracted data points calculated from at least three independent experiments. The exchange pattern remained the same in all three control conditions and deviations in the fraction exchanged did not exceed 10%.
(TIF)

Figure S2 **Isotopic envelopes show a shift in time.** Comparison of the isotopic envelopes for two peptides, SASEL (left panel) and PRVWEPVPLEE (right panel), after 10 seconds of exchange. Typical traces at appropriate m/z and ion mobility drift time regions represent (from lowest to highest trace): the signal for the in-exchange control experiment, dimRAGE, exRAGE, and the out-exchange control experiment. The weighted average masses are also given. The differences in weighted average masses between exRAGE and dimRAGE were reproducible in repeated experiments, as illustrated by the error bars in Figure 6.
(TIF)

Figure S3 **Solvent accessible surface for monomeric and dimeric RAGE.** Differences in the solvent accessible surface between the dimer (Figure 7) and monomer of the same structure as calculated for residues along the RAGE sequence of both monomers, marked by blue and purple traces. Peptide sequences in the protected regions are also shown.
(TIF)

Figure S4 **Binding surfaces of the exRAGE dimer.** The concave binding surface of the C2 symmetry dimer from Figure **7** is shown with visualization of positively (blue) and negatively (red) charged residues.
(TIF)

Table S1 **Related to Figure 4: The peptic peptide sequences of exRAGE.** The list was obtained from LC-MS-MS/MS analysis of the non-deuterated sample analyzed by the PLGS program. Peptides are sorted according to their position in the protein sequence. mass = molecular mass, rt = retention time.
(DOC)

Text S1 **Supplemental Experimental Procedures.**
(DOC)
