# Supplementary figures and images for: Oligomerization Interface of RAGE Receptor Revealed by MS-Monitored Hydrogen Deuterium Exchange
Source: PLoS One. 2013 Oct 1;8(10):e76353. doi: 10.1371/journal.pone.0076353 (PMC3788119; doi:10.1371/journal.pone.0076353)

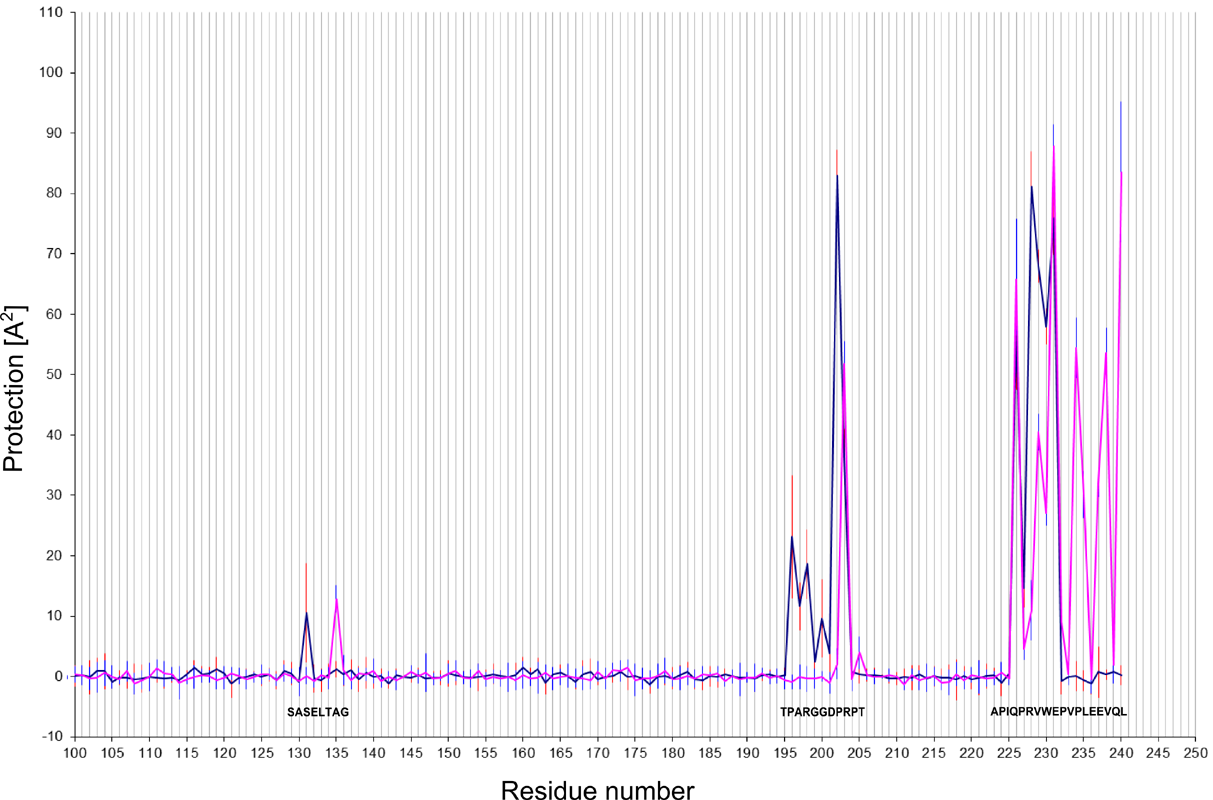

Supplement: Figure S3 — Solvent accessible surface for monomeric and dimeric RAGE. Differences in the solvent accessible surface between the dimer (Figure 7) and monomer of the same structure as calculated for residues along the RAGE sequence of both monomers, marked by blue and purple traces. Peptide sequences in the protected regions are also shown. (TIF) [file pone.0076353.s004.tif]
