# Supplementary material for: Oligomerization Interface of RAGE Receptor Revealed by MS-Monitored Hydrogen Deuterium Exchange
Source: PLoS One. 2013 Oct 1;8(10):e76353. doi: 10.1371/journal.pone.0076353 (PMC3788119; doi:10.1371/journal.pone.0076353)
Supplement: Table S1 — Related to Figure 4: The peptic peptide sequences of exRAGE. The list was obtained from LC-MS-MS/MS analysis of the non-deuterated sample analyzed by the PLGS program. Peptides are sorted according to their position in the protein sequence. mass = molecular mass, rt = retention time. (DOC) [file pone.0076353.s006.doc]

**Table S1 The peptic peptide sequences of exRAGE.**

| Peptide sequence | Position in sequence | | mass | rt |
| --- | --- | --- | --- | --- |
|  | start | end |  |  |
| AQNITA | 23 | 28 | 617.3253 | 2.55 |
| AQNITARIGEPLVL | 23 | 36 | 1494.8639 | 5.91 |
| EWKLNTGRTEA | 50 | 60 | 1304.6593 | 4.40 |
| WKVLSPQGGGPWDSV | 61 | 75 | 1612.8118 | 6.23 |
| WKVLSPQGGGPWDSVA | 61 | 76 | 1683.8489 | 6.06 |
| ARVLPNGSL | 76 | 84 | 926.5418 | 4.51 |
| ARVLPNGSLFL | 76 | 86 | 1186.6943 | 6.06 |
| RVLPNGSL | 77 | 84 | 855.5047 | 4.43 |
| RVLPNGSLF | 77 | 85 | 1002.5731 | 5.53 |
| RVLPNGSLFL | 77 | 86 | 1115.6572 | 6.07 |
| PNGSL | 80 | 84 | 487.2511 | 4.41 |
| PAVGIQDE | 87 | 94 | 828.4098 | 4.12 |
| PAVGIQDEGI | 87 | 96 | 998.5153 | 5.16 |
| AMNRNGKETKSNYRV | 101 | 115 | 1767.8919 | 5.41 |
| RVRV | 114 | 117 | 529.3569 | 2.47 |
| RVRVYQIPGKPEIVD | 114 | 128 | 1769.0068 | 4.41 |
| RVRVYQIPGKPEIVDS | 114 | 129 | 1856.0389 | 4.30 |
| RVRVYQIPGKPEIVDSASEL | 114 | 133 | 2256.2347 | 4.91 |
| YQIPGKPEIVD | 118 | 128 | 1258.6678 | 4.69 |
| YQIPGKPEIVDS | 118 | 129 | 1345.6998 | 4.61 |
| YQIPGKPEIVDSASE | 118 | 132 | 1632.8115 | 4.63 |
| YQIPGKPEIVDSASEL | 118 | 133 | 1745.8956 | 5.34 |
| SASEL | 129 | 133 | 506.2457 | 3.99 |
| ASEL | 130 | 133 | 419.2136 | 3.90 |
| SWHLDGKPLVPNEKGVS | 156 | 172 | 1862.9759 | 4.75 |
| HLDGKPLVPNEKGVS | 158 | 172 | 1589.8646 | 2.61 |
| DGKPLVPNEKGVS | 160 | 172 | 1589.8646 | 3.02 |
| KPLVPNEKGVSVKEQTR | 162 | 178 | 1909.0865 | 4.88 |
| PNEKGVS | 166 | 172 | 730.3730 | 2.71 |
| VKEQTRRHPETGL | 173 | 185 | 1550.8398 | 2.48 |
| VKEQTRRHPETGLF | 173 | 186 | 1697.9082 | 4.09 |
| FTLQSE | 186 | 191 | 724.3512 | 4.82 |
| FTLQSEL | 186 | 192 | 837.4353 | 5.97 |
| TLQSEL | 187 | 192 | 690.3668 | 5.04 |
| MVTPARGGDPRPTF | 193 | 206 | 1501.7580 | 4.35 |
| CSFSPGLPRHRALRTAPIQ | 208 | 226 | 2107.1342 | 4.80 |
| PRVWEPVPLE | 227 | 236 | 1221.6626 | 5.65 |
| PRVWEPVPLEE | 227 | 237 | 1350.7052 | 5.68 |
| VVEPEGGAVAPGGTVT | 241 | 256 | 1577.8322 | 5.82 |
| APGGTVT | 250 | 256 | 1439.7377 | 4.07 |
| IHWMKDGVPLPLPPSPVL | 269 | 286 | 1996.1089 | 4.88 |
| HWMKDGVPLPLPPSPVL | 270 | 286 | 1883.0248 | 5.86 |
| MKDGVPLPLPPSPVL | 272 | 286 | 1559.8866 | 6.00 |
| PEIGPQDQGT | 289 | 298 | 1041.4847 | 3.79 |
| VATHSSHGPQ | 302 | 311 | 1020.4857 | 5.13 |
| VSIS | 316 | 319 | 405.2344 | 3.35 |
