# Supplementary material for: Oligomerization Interface of RAGE Receptor Revealed by MS-Monitored Hydrogen Deuterium Exchange
Source: PLoS One. 2013 Oct 1;8(10):e76353. doi: 10.1371/journal.pone.0076353 (PMC3788119; doi:10.1371/journal.pone.0076353)
Supplement: Text S1 — Supplemental Experimental Procedures. (DOC) [file pone.0076353.s007.doc]

**Text S1 Supplemental Experimental Procedures.**

**Protein expression and purification*.***

The exRAGE protein and its variant YexRAGE were over-expressed in bacteria. The expression construct containing a C-terminal His-tag was a gift from Dr. Rosemarie Wilton, Argonne National Laboratory, IL, USA. YexRAGE was obtained by a point mutation at position 340 that changed threonine to tyrosine. For protein over-expression, a glycerol stock of JM83 cells transformed with plasmid encoding exRAGE was inoculated into 30 ml of 2xYT medium with 100 µg/mL carbenicillin. The bacteria were grown overnight at 37°C with shaking at 250 rpm. A 5 mL aliquot of the resultant culture was later inoculated into 1 L of 2×YT medium with 100 µg/mL carbenicillin and grown at 37°C with shaking at 250 rpm for approximately 5 h to an OD_600_ of 1-1.2. Then, the temperature of the culture was changed to 28°C, the speed of shaking was changed to 145 rpm, and the culture was grown to an OD_600_ of 1.5-1.6 over 2 h. Protein expression was induced by adding IPTG at a final concentration of 1 mM. The culture was incubated overnight and the cells harvested by centrifugation.

Cells from 1 L of culture were re-suspended in 100 mL TES buffer (20 mM Tris-HCl, 0.5 mM EDTA, and 0.5 M sucrose, pH 8.0) with 60 µg/mL of lysozyme (Sigma, St. Louis, MO, USA), 0.2 mM PMSF (Sigma, St. Louis, MO, USA), and 10 µg/mL deoxyribonuclease I (Sigma, St. Louis, MO, USA). The suspension was gently agitated for 1 h at 4°C and then centrifuged at 6000 rpm for 20 min at 4°C. The supernatant was dialyzed twice against 2 L of chromatography buffer (20 mM Tris-HCl, 300 mM NaCl, and 10 mM imidazole, pH 8.0), first for 2 h and then overnight at 4°C, and centrifuged at 6000 rpm for 20 min at 4°C. The supernatant was then mixed with 4 mL of Ni-NTA resin (Qiagen, Hilden, Germany) equilibrated with chromatography buffer and washed with 10 bed volumes of chromatography buffer. The protein was eluted using elution buffer (20 mM Tris-HCl, 300 mM NaCl, 250 mM imidazole, pH 8.0) and the OD_280_ monitored for all fractions. Fractions containing the protein were combined and dialyzed twice against dialysis buffer (20 mM Tris-HCl, 150 mM NaCl, 1 mM EDTA, pH 7.2) as described previously. Subsequently, the fast protein liquid chromatography purification step was performed using the Hi-Trap SP (General Electric – Healthcare, Fairfield, CT, USA) column and a buffer gradient: A (50 mM Tris-HCl and 50 mM NaCl, pH 7.2) and B (50 mM Tris-HCl and 1 M NaCl, pH 7.2). The protein eluted at 80% buffer B. The typical preparation allowed 1 mL of exRAGE at a concentration of 150-300 µM to be obtained. Protein concentration was measured spectrophotometrically (Cary 50 Bio, Varian, Palo Alto) at 280 nm assuming a molar extinction coefficient of 38,960 M^-1^cm^-1^.

**MALDI-TOF mass spectrometric analysis.**

The molecular mass of exRAGE subjected to HRP/H_2_O_2_ treatment was verified by matrix-assisted laser desorption**/**ionization time-of-flight (MALDI-TOF/TOF ultrafleXtreme, Bruker) mass spectrometry. The mass spectrometer parameters were the manufacturer’s recommended settings adjusted for optimal mass performance.

The molecular mass of exRAGE subjected to HRP/H_2_O_2_ treatment was verified by matrix-assisted laser desorption**/**ionization time-of-flight (MALDI-TOF/TOF ultrafleXtreme, Bruker) mass spectrometry. The mass spectrometer parameters were the manufacturer’s recommended settings adjusted for optimal mass performance.

The laser repetition rate was set at 1000 Hz frequency with 800 laser shots, the laser attenuation range was 70%, and a delay time of 300 ns. Linear ion modes were used for positive ion detection and mass spectra were acquired in a range of 30-150 kDa. Samples were placed on a stainless steel plate, mixed with matrix (sinapinic acid (Fluka) prepared in 80% (v/v) acetonitrile/0.3% trifluoroacetic acid) at a ratio of 1:1 and air-dried prior to analysis. External calibration was performed with bovine albumin (Sigma) using the m/z value of 66,431 Da for the mono-charged ion and 33,216 Da for the doubly-charged ion. The values expressed are the average mass and correspond to the [M+H]^+^ ion. The MALDI-MS spectral data were processed using FlexAnalysis 2.0 software from Bruker.

**Dot blot assay.**

Samples of BSA (Sigma) and peptide Aβ1-40 (obtained by over-expression [1]) were serially diluted with TBS and applied to nitrocellulose membrane (0.45 μm; Serva) using a BioDot filtration apparatus (Bio-Rad). The membrane was removed from the apparatus, blocked at room temperature for 2 h in a blocking buffer (Sigma), transferred to TBS-T containing 0.5 μM exRAGE or dimRAGE, and incubated overnight at 4°C. The membrane was washed five times for 10 min with TBS-T and incubated with Anti-His[HRP] Monoclonal Antibody 1:5000 (Genscript) for 1 h at room temperature, followed by washing. Immobilized RAGE was detected by chemiluminescence using ECL Western Blotting Detection Reagents (GE Healthcare). Spot intensities were quantitated using GelQuant software.

**Hydrogen deuterium exchange.**

The first step of the analysis established a list of peptic RAGE peptides using non-deuterated sample. A 5 µL aliquot of protein stock (at least 20 µM in 50 mM Tris, pH 7.2, and 800 mM NaCl) was diluted 10 times by adding 45 µL of 50 mM Tris, pH 8, and 83 mM NaCl (H_2_O Reaction buffer) to maintain the final NaCl concentration of 150 mM. Next, the sample was acidified by mixing with 10 µL of 2 M glycine buffer, pH 2.5 (H_2_O Stop Buffer). Also, 2 µL TCEP (0.4 g/mL) was added prior to LC-MS analysis to reduce disulfide bonds. The sample was subjected to online pepsin digestion using a 2.1 mm x 30 mm immobilized pepsin resin column (Porozyme, ABI, Foster City, CA) with 200 µL/min flow of 0.07% formic acid in water as a mobile phase. Digested peptides were passed directly to the 2.1 mm x 5 mm C18 trapping column (Acquity BEH C18 VanGuard precolumn, 1.7 µm resin, Waters, Milford, MA). Trapped peptides were eluted onto a reverse phase column (Acquity UPLC BEH C18 column, 1.0 x 100 mm, 1.7 µm resin, Waters, Milford, MA) using a 6% to 40% gradient of acetonitrile in 0.1% formic acid (flow 40 µL/min) supplied by a nanoACQUITY Binary Solvent Manager. The total time of a single run was 13.5 minutes. All fluidics, valves, and columns were maintained at 0.5°C using the HDX Manager (Waters, Milford, MA) beside the pepsin digestion column, which was kept at 20°C inside the temperature-controlled digestion column compartment of the HDX manager. The C18 column outlet was coupled directly to the ion source of a SYNAPT G2 HDMS mass spectrometer (Waters, Milford, MA) working in Ion Mobility mode. Lock mass was activated and carried out using leucine-enkephalin (Sigma). For protein identification, mass spectra were acquired in MS^E^ mode over the m/z range of 50 – 2000. The spectrometer parameters were as follows: ESI positive mode, capillary voltage 3 kV, sampling cone voltage 40 V, extraction cone voltage 4 V, source temperature 80°C, desolvation temperature 175°C, and desolvation gas flow 600 L/h. The spectrometer was calibrated on a weekly basis using standard calibration solutions.

Peptides were identified using ProteinLynx Global Server software (Waters, Milford, MA). The list of identified peptides containing peptide m/z, charge, retention time, and ion mobility drift time was passed to the DynamX hydrogen deuterium data analysis program (Waters, Milford, MA).

Hydrogen-deuterium exchange experiments were carried out as described for non-deuterated samples with the Reaction buffer prepared using D_2_O (99.8% Armar Chemicals, Switzerland), in which the pH_read_ (uncorrected meter reading) was adjusted using DCl or NaOD (Sigma). After mixing 5 µL of protein stock with 45 µL of D_2_O r The notation was edited for consistency, or 20 min) at room temperature. The exchange was quenched by reducing the pH_read_ to 2.5 by adding the reaction mixture into an Eppendorf tube containing Stop buffer (2 M glycine buffer, pH_read_ 2.5) cooled on ice. A total of 2 µL TCEP (0.4 g/mL) was added prior to LC-MS analysis to reduce disulfide bonds. Immediately after quenching, the sample was manually injected into the nanoACQUITY (Waters, Milford, MA) UPLC system. Further pepsin digestion, LC, and MS analysis was carried out exactly as described for the non-deuterated sample.

Two control experiments were carried out to take into account in-exchange and out-exchange artefacts as described previously [2]. Briefly, to assess minimum exchange (in-exchange control) D_2_O Reaction buffer was added to Stop buffer cooled on ice prior to protein stock addition and the mixture immediately subjected to pepsin digestion and LC-MS analysis as described above. The deuteration level in the in-exchange experiment was calculated using DynamX and denoted as 0% exchange (M_ex_^0^). For out-exchange analysis, the 5 µL protein stock was mixed with 55 µL of D_2_O Reaction buffer, incubated overnight, mixed with Stop buffer, and analyzed as described above. The deuteration level in the out-exchange experiment was calculated and denoted as 100% exchange (M_ex_^100^).

The above experimental scheme, consisting of in-exchange and out-exchange controls and the HDex experiment with the sample incubated for different times in D_2_O, enabled us to obtain the same set of fragments in control experiments and the HDex experiment. All HDex experiments and control experiments were repeated at least three times, and the final results represent a mean from these experiments.

**Experimental References**

1. Kłoniecki M, Jabłonowska A, Poznański J, Langridge J, Hughes C, et al. (2011) Ion Mobility Separation Coupled with MS Detects Two Structural States of Alzheimer’s Disease Aβ1–40 Peptide Oligomers. Journal of Molecular Biology 407: 110–124. doi:10.1016/j.jmb.2011.01.012.

2. Kupniewska-Kozak A, Gospodarska E, Dadlez M (2010) Intertwined Structured and Unstructured Regions of exRAGE Identified by Monitoring Hydrogen–Deuterium Exchange. Journal of Molecular Biology 403: 52–65. doi:10.1016/j.jmb.2010.08.027.
